# Diagnostic and Predictive Value of CD133‐Positive Circulating Tumor Cells as an Indicator of Pathological High‐Risk Factors for Stage I Non‐Small Cell Lung Cancer

**DOI:** 10.1002/cam4.71303

**Published:** 2025-10-29

**Authors:** Huandong Huo, Xiaoli Zhang, Qi Zhang, Zhuoheng Lv, Peipei Xie, Kaitai Zhang, Wen Zhang, Yousheng Mao

**Affiliations:** ^1^ Department of Thoracic Surgery, National Cancer Center/National Clinical Research Center for Cancer/Cancer Hospital Chinese Academy of Medical Sciences and Peking Union Medical College Beijing China; ^2^ State Key Laboratory of Molecular Oncology, Department of Etiology and Carcinogenesis, National Cancer Center/National Clinical Research Center for Cancer/Cancer Hospital Chinese Academy of Medical Sciences and Peking Union Medical College Beijing China

**Keywords:** CD133, circulating tumor cells, diagnosis model, non‐small cell lung cancer, pathological high‐risk factors

## Abstract

**Background:**

The noninvasive prediction of pathological high‐risk factors in stage I NSCLC patients before surgery remains an area for further investigation, and circulating stem cancer cells are a potential predictive diagnostic indicator. Identifying these factors preoperatively can guide treatment decisions and may improve outcomes. Here, we aim to explore CD133‐positive circulating tumor cells (CTCs) in stage I patients of NSCLC to predict pathological high‐risk factors of patients, thereby aiding clinical decision‐making.

**Methods:**

A total of 192 Stage I NSCLC patients were finally enrolled and underwent surgical intervention. We assessed the level of CD133‐positive CTCs by employing the telomerase reverse Transcriptase‐Based CTC Detection method. The Least Absolute Shrinkage and Selection Operator method (LASSO) and logistic regression were used to analyze the association between CD133‐positive CTCs with pathological high‐risk factors and construct a diagnostic model.

**Results:**

Among all the enrolled patients, postoperative pathology confirmed that 12 patients had pathological high‐risk factors, while 180 patients had no high‐risk factors. The median count of CD133‐positive CTCs before surgery in patients with high‐risk factors was recorded at 1.58 ± 1.83, significantly higher than 0.767 ± 1.13 observed in the group without high‐risk factors (*p* = 0.048). Following feature selection by LASSO regression and multivariate logistic regression, it was determined that CD133‐positive CTCs, CT imaging nodule features, and elevated CEA levels can be combined as diagnostic indicators for pathological high‐risk factors in NSCLC patients. The ROC curve derived from internal bootstrap validation, alongside the calibration and DCA plots, affirmed the model's accuracy and predictive capability. After follow‐up, we found that patients without high‐risk pathological factors showed better PFS (*p* = 0.044).

**Conclusions:**

This study indicated that CD133‐positive CTCs were associated with pathological high‐risk factors, and the detection of CD133‐positive CTCs may assist treatment decision‐making for patients of NSCLC.

## Graphical abstract

This study explores the predictive value of combining CD133‐positive circulating tumor cells in a diagnostic model for identifying high‐risk pathological factors in stage I NSCLC. Identifying these factors preoperatively can guide treatment decisions and may improve outcomes. By integrating CD133‐positive CTCs counts with CT imaging features and CEA levels, the model supports preoperative clinical decisions, demonstrating strong predictive accuracy.

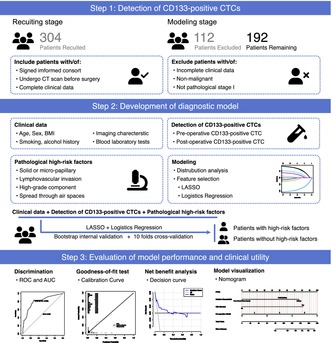

## 1 Introduction

Lung cancer, the foremost cause of cancer‐related deaths worldwide, has a high morbidity and mortality rate, with non‐small cell lung cancer (NSCLC) accounting for more than 89% of cases [1, 2]. Early‐stage NSCLC patients generally exhibit favorable post‐surgery outcomes, with a 5‐year survival rate exceeding 80% [3]. As the stage advances, patient survival trends downwards, with stage Ib patients experiencing a reduced 5‐year survival rate of 69% [3]. Numerous studies have examined this issue from the perspective of pathological risk factors to explore the reasons behind the relatively poorer survival rate among patients with stage Ib NSCLC. The findings of these studies highlight the importance of additional treatment for these patients [4, 5, 6]. These pathological risk factors include T stage, histologic subtype, especially high‐grade subtypes [7, 8, 9, 10], microscopic vessel invasion, lymphovascular invasion (LVI), visceral pleural invasion (VPI) [11], spread through air spaces (STAS) [12], solid or micropapillary components, and vascular invasion or airway spread [13, 14, 15]. Notably, among patients with similar pathological high‐risk factors, patients who underwent more extensive resections, such as lobectomy or pneumonectomy, demonstrated better survival rates compared to those who underwent partial resections [14]. However, current comprehensive studies on the effectiveness of noninvasive presurgical techniques in identifying these pathological risk factors remain limited.

Given that cancer stem cells (CSCs) have been demonstrated to facilitate immune evasion, enhance therapeutic resistance, and significantly increase the risk of disease recurrence [16], they have received increasing attention as potential targets for therapeutic intervention [17]. In studies of prostate cancer and breast cancer, CD133, also known as Prominin 1, serves as a key surface marker for identifying tumor cells with stem‐like properties, and CD133‐positive circulating tumor cells (CTCs) have shown stem‐like potential [18, 19, 20]. These findings further emphasize the critical role of CSCs in cancer progression. Given the role of CSCs in promoting aggression and recurrence in other cancers, we hypothesized that CD133‐positive CTCs might also be associated with high‐risk pathological features in NSCLC.

Based on our previous research results, the telomerase reverse transcriptase‐based CTC assay (TBCD) has demonstrated stable performance in distinguishing benign and malignant pulmonary nodules [21]. This approach offers significant clinical application prospects due to its noninvasive, early diagnostic capabilities and potential for early recurrence monitoring. Building upon this foundation, the current study aims to explore the potential diagnostic value of CD133‐positive CTCs in a preoperative noninvasive assessment approach, particularly focusing on their predictive ability for pathological high‐risk factors. Through this study, we expect to further uncover the clinical application potential of CD133‐positive CTCs as a biomarker in stage I patients of NSCLC, thereby providing a scientific basis for optimizing treatment strategies and improving patient survival rates.

## 2 Materials and Methods

### 2.1 Study Design

**FIGURE 1:**
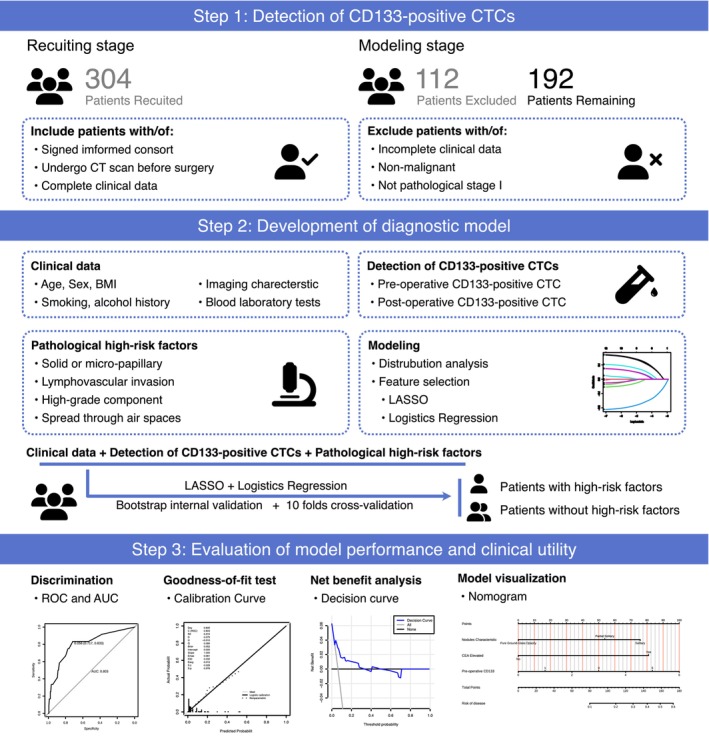
Workflow of our study. The whole process ranged from patient inclusion and exclusion to model construction.

The graphical abstract (Figure 1) outlines the logical workflow of the study, encompassing the study design, analytical pipeline, and principal findings. Between January 2021 and June 2023, 304 lung cancer patients were enrolled at the Cancer Hospital of the Chinese Academy of Medical Sciences, among whom 192 patients were included in this retrospective study. The inclusion criteria were: (1) receipt of surgical resection; (2) definitive diagnosis of stage I NSCLC; and (3) availability of preoperative CT imaging and complete postoperative pathological reports. Ethical approval for the study was granted by the Ethics Committee (approval number: No. 21/093‐2764), and all patients provided informed consent to participate in the study.

The baseline clinical information collected included sex, age, cigarette smoking history, weight, height, and family history of cancer. Preoperative CT scan recorded radiological features, such as tumor size, morphology, location, radiologic lymph node metastasis, atelectasis, and pleural effusion. The key pathologic parameters, including tumor grade, histologic subtype, vascular invasion, perineural invasion, and micropapillary components, were extracted from postoperative pathology reports. Following the current National Comprehensive Cancer Network (NCCN) guidelines, pathological high‐risk patients were defined as patients with one or more of the following characteristics: (1) microscopic vascular invasion; (2) VPI; (3) perineural invasion; (4) pleural invasion (Hammer's classification stage > 1); (5) STAS; or (6) a proportion of high‐grade components > 20%. Therefore, we stratified into groups for further analysis according to the postoperative pathological diagnosis results and strictly based on the existence of the above pathological high‐risk factors.

### 2.2 Blood Sampling and CD133‐Positive CTCs Enrichment

In the perioperative period of each patient, blood samples were collected both preoperatively and postoperatively for CTC detection. In brief, the preoperative blood samples were primarily collected 1 day before surgery; if this was not possible due to personal or surgical scheduling reasons, the samples were collected 3–4 days in advance. A small number of patients had their samples collected on the morning of the surgery, but always prior to the start of the operation. Postoperative blood samples were mainly collected between the 3rd and 5th day after surgery, in order to ensure the patient had generally recovered and the testing conditions were relatively stable.

The detection method was based on our previous research protocol [21], but was modified by incorporating an anti‐CD133 antibody into the antibody panel to detect CD133‐positive CTCs. Briefly, 4 mL of peripheral blood was collected in ethylene diamine tetra‐acetic (EDTA) acid anticoagulant tubes. Following centrifugation, the supernatant plasma was removed. The lower blood cells were lysed and subsequently resuspended in serum‐free medium. The resuspension was transduced with oHSV1‐hTERTp‐GFP (MOI = 1). After incubation for 24 h, transduced cells were collected and stained with APC‐Cyanine7 anti‐human CD45 antibody (Clone: HI30) and APC anti‐human CD133 antibody (Clone: clone7).

### 2.3 Identification and Analysis of CTCs by Imaging Flow Cytometry

Identification and quantification of CTCs were performed using an imaging flow cytometer (Amnis ImageStream MK II, Luminex). After washing with phosphate‐buffered saline (PBS), cells were analyzed on this instrument. Subsequently, the imaging data were analyzed using IDEAS software (version 6.2). CTCs were defined as CD45^−^/GFP^+^ events, and CD133‐positive CTCs were defined as CD45^−^/GFP^+^/CD133^+^ events.

### 2.4 Model Evaluation and Validation

Key variables associated with pathological high‐risk factors were identified from all clinical characteristics using least absolute shrinkage and selection operator (LASSO) regression. The optimal λ value of LASSO regression was determined by conducting cross‐validation. Subsequently, the variables filtered by LASSO regression were incorporated in a multivariate logistic regression process to establish a diagnostic model for the patients with pathological high‐risk.

The performance of the model was comprehensively evaluated. Discrimination was assessed by the receiver operating characteristic curve (ROC) and its area under the ROC curve (AUC). Calibration was evaluated using a calibration plot to compare predicted probabilities against observed outcomes. Clinical utility was quantified by decision curve analysis (DCA) to estimate the net benefit across a range of threshold probabilities. Finally, the model's internal validity and stability were tested using bootstrap validation with 1000 resamples.

### 2.5 Nomogram Construction

A nomogram was developed using the results of a multivariate logistic regression model to visually quantify the impact of each predictive factor on the risk of pathological outcomes. The nomogram integrates CD133‐positive CTCs and other clinical variables, allowing individualized risk assessment for patients. This tool can assist clinicians in making personalized treatment decisions.

### 2.6 Statistical Analysis

All statistical analyses, model construction, and graph plotting were performed using R software (version 4.4.1; The R Project for Statistical Computing). The Shapiro–Wilk test and Levene's test were used to assess normality of data and homogeneity of variance. Data with gaussian distribution were displayed as mean ± SD. Comparisons between groups were conducted using the independent samples *t*‐test. For non‐normally distributed data, they were presented as M (P25, P75), and group comparisons were performed using the Wilcoxon rank‐sum test. Categorical variables were represented by numbers (%). LASSO regression with 10‐fold cross‐validation was performed using the glmnet package, while multivariate logistic regression and nomogram models were constructed using the rms package. The receiver operating characteristic (ROC) curves, calibration curves, and decision curve analyses were plotted using the pROC, rms, ggDCA, and ggplot2 package, respectively. Overall survival (OS) was defined as the duration from surgery to death or the last follow‐up. Disease‐free survival (DFS) is defined as the time from surgery to either radiological or clinical progression, or to the last contact if no progression is observed. Survival analysis was performed using Kaplan–Meier methods. Differences between groups were evaluated by Log‐rank test. Statistical tests were two‐sided unless specified otherwise, and a *p*‐value < 0.05 was considered statistically significant.

## 3 Results

### 3.1 Patient Characteristics and Clinical Pathological Features

This study included an overall total of 192 patients, categorized into two different groups according to the existence of pathological high‐risk factors: 180 patients without pathological high‐risk factors and 12 patients with pathological high‐risk factors. The demographic and baseline characteristics of the patients are detailed in Table 1. The key baseline characteristics are summarized as follows.

**TABLE 1:** Patient demographic and baseline characteristics of patients.

	Normal group^a^ (*N* = 180)	High‐risk group^b^ (*N* = 12)	*p*
Age			
Mean (SD)	60.9 (9.76)	63.4 (8.11)	0.508
Sex			
Female	120 (66.7%)	7 (58.3%)	0.783
Male	60 (33.3%)	5 (41.7%)	
Preoperative CD133			
Mean (SD)	0.767 (1.13)	1.58 (1.56)	0.048
Median [min, max]	0 [0, 6.00]	1.00 [0, 4.00]	
Postoperative CD133			
Mean (SD)	1.22 (2.93)	1.58 (1.83)	0.315
Median [min, max]	0 [0, 35.0]	1.00 [0, 5.00]	
Smoking history			
No	143 (79.4%)	8 (66.7%)	0.288
Yes	37 (20.6%)	4 (33.3%)	
BMI			
Mean (SD)	23.6 (3.07)	22.8 (2.20)	0.371
Median [min, max]	23.5 [17.3, 36.2]	22.3 [19.5, 27.1]	
Missing	2 (1.1%)	0 (0%)	
Pathology type			
Adenocarcinoma	113 (62.1%)	11 (91.7%)	0.265
Squamous cell carcinoma	54 (29.7%)	1 (8.3%)	
Early cancerous or noninvasive lesions	2 (1.1%)	0 (0%)	
Others^c^	13 (7.1%)	0 (0%)	
Perineural invasion			
No	177 (98.3%)	12 (100%)	1
Yes	3 (1.7%)	0 (0%)	
Vascular invasion			
No	175 (97.2%)	10 (83.3%)	0.0632
Yes	5 (2.8%)	2 (16.7%)	
Involvement of the lobes bronchi and below			
No	176 (97.8%)	12 (100%)	1
Yes	4 (2.2%)	0 (0%)	
Cavity dissemination			
No	167 (92.8%)	11 (91.7%)	1
Yes	13 (7.2%)	1 (8.3%)	
Pleural invasion			
No	180 (100%)	1 (8.3%)	< 0.001
Yes	0 (0%)	11 (91.7%)	
High‐grade structures			
Mean (SD)	0.0539 (0.184)	0.0375 (0.0377)	0.0149
Median [min, max]	0 [0, 1.60]	0.0500 [0, 0.100]	
Elevated Cyfra21‐1			
No	159 (88.3%)	9 (75.0%)	0.177
Yes	21 (11.7%)	3 (25.0%)	
Elevated NSE			
No	161 (89.4%)	9 (75.0%)	0.145
Yes	19 (10.6%)	3 (25.0%)	
Elevated SCC			
No	175 (97.2%)	11 (91.7%)	0.325
Yes	5 (2.8%)	1 (8.3%)	
Elevated CEA			
No	174 (96.7%)	10 (83.3%)	0.0814
Yes	6 (3.3%)	2 (16.7%)	
Elevated ProGRP			
No	178 (98.9%)	12 (100%)	1
Yes	2 (1.1%)	0 (0%)	
Nodules counts			
1	146 (81.1%)	10 (83.3%)	1
2	34 (18.9%)	2 (16.7%)	
Nodule features			
Pure ground glass nodules	64 (35.6%)	1 (8.3%)	0.141
Part‐solid nodules	70 (38.9%)	6 (50.0%)	
Solid nodules	46 (25.5%)	5 (41.7%)	

^a^ Normal group: Patients without pathological high‐risk factors.

^b^ High‐risk group: Patients with pathological high‐risk factors.

^c^ Other: These other pathological types mainly include: adenosquamous carcinoma, large cell lung carcinoma, and a small amount of sarcomatoid carcinoma.

The group of patients without pathological high‐risk factors consisted of 120 females (66.7%) and 60 males (33.3%), while the group of patients with pathological high‐risk factors included 7 females (58.3%) and 5 males (41.7%), with no significant gender difference between the groups (*p* = 0.315). The mean age was 60.9 years in the group of patients without pathological high‐risk factors and 63.4 years in the group of patients with pathological high‐risk factors, with no significant difference (*p* = 0.508). In terms of smoking history, 79.4% of patients in the group of patients without pathological high‐risk factors and 66.7% in the group of patients with pathological high‐risk factors were nonsmokers, with no significant difference observed (*p* = 0.288). The body mass index (BMI) was similar (*p* = 0.371).

With respect to pathological features, there were no significant differences in perineural invasion (*p* = 1), vascular invasion (*p* = 0.0632), lobar bronchial invasion (*p* = 1), or cavity dissemination (*p* = 1) between the groups. However, pleural invasion was significantly more common in patients with pathological high‐risk factors (91.7%) compared to patients without pathological high‐risk factors (*p* < 0.001).

For tumor markers, elevated Cyfra21‐1 was observed in 11.7% of patients without pathological high‐risk factors and in 25.0% of patients with high‐risk factors. Similarly, elevated neuron‐specific enolase (NSE) levels were found in 10.6% of patients without high‐risk factors compared to 25.0% in those with high‐risk factors; however, these differences did not reach statistical significance. No significant differences were observed in levels of squamous cell carcinoma (SCC) or pro‐gastrin‐releasing peptide (ProGRP) between the two groups. Notably, elevated carcinoembryonic antigen (CEA) levels were more frequent in patients with pathological high‐risk factors (16.7%) than in those without (3.3%), yet this difference did not reach statistical significance. In addition, nodule characteristics were similar between the groups, with no significant differences in the number of nodules or the proportion of pure ground‐glass, part‐solid, or solid nodules (*p* > 0.05).

### 3.2 TBCD Combined With Anti‐CD133 Antibody Is a Feasible Method for Detecting CD133‐Positive CTCs

**FIGURE 2:**
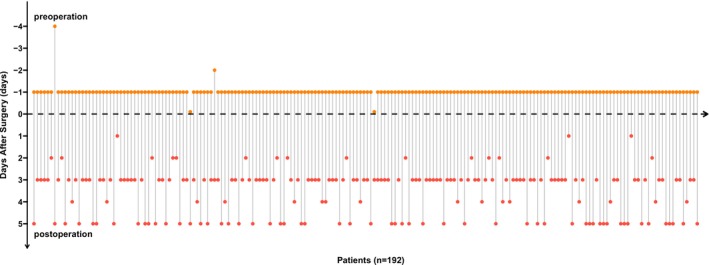
Overview of sample collection time points. The lollipop plot illustrates the data from sample collection time points. Each data point represents a specific measurement or result obtained at various intervals both pre‐ and post‐surgery. The x‐axis denotes “Days After Surgery,” with values extending from negative to positive, signifying that samples were collected both prior to (negative days) and following (positive days) the surgical procedure. The preoperative blood samples marked as day 0 were collected on the day of surgery, but before the surgical procedure.

**FIGURE 3:**
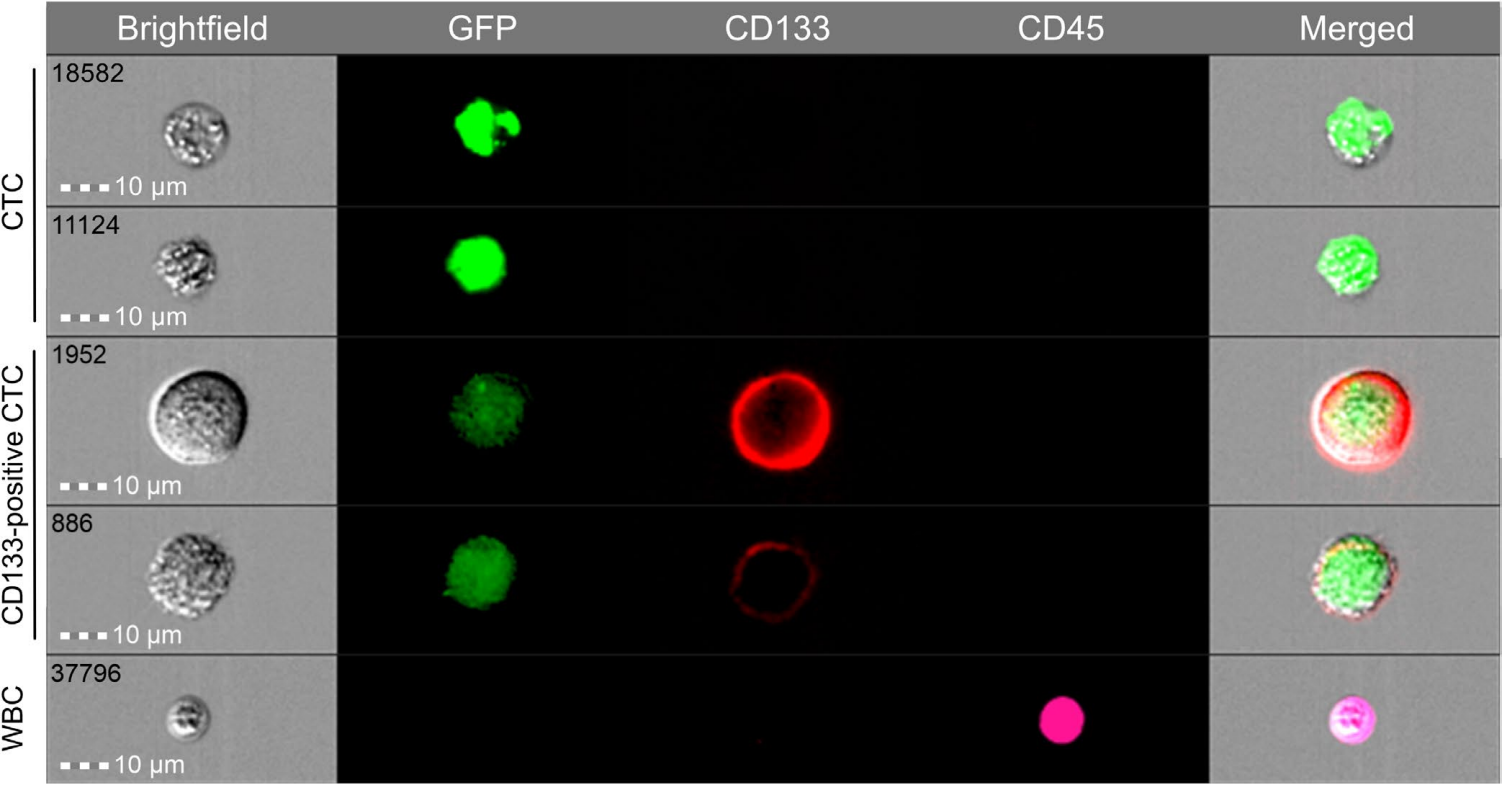
Images of CTCs detection acquired by microscopy after selecting the cells using imaging flow cytometry. Images of representative CD133‐positive CTC, CTC, and WBC acquired using FlowSight, an imaging flow cytometry approach, showcase bright‐field, TERT‐GFP, CD133‐APC, and CD45‐APC/Cyanine7 channels. CTC was defined as CD45^−^/GFP^+^, CD133^+^ CTC was defined as CD45^−^/GFP^+^/CD133^+^, and WBC as CD45^+^/GFP^−^.

In our previous study [21], the use of the TBCD showed its stability in differentiating between benign and malignant lung nodules. To clearly illustrate the distribution of sampling times at each time point, we visualized the blood sample collection times for all 192 patients (Figure 2). To further detect CD133‐positive CTCs in the peripheral blood, we employed TBCD combined with the anti‐CD133 antibody (Figure 3). Flow imaging technology was used to visualize CTC, CD133‐positive CTC, and white blood cells (WBCs) captured from the peripheral blood of patients. The results illustrated that, compared with WBCs, CTCs were larger in size and had relatively irregular shapes. At the molecular phenotype level, CTCs exhibited high GFP expression (TERT^+^) and deficient expression of leukocyte common antigen (CD45), whereas CD133‐positive CTCs presented high GFP expression (TERT^+^) and CD133 expression, without CD45 expression. Conversely, all WBCs expressed high levels of CD45 and were identified as CD45^+^/TERT^−^. All 192 patients in this cohort successfully completed CTC and CD133 marker detection.

**FIGURE 4:**
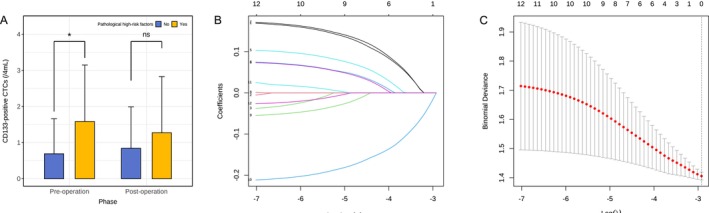
Comparison of CD133‐positive CTC levels and LASSO regression analysis. (A) Preoperative CD133‐positive CTC levels between groups with and without pathological high‐risk factors show a significant difference (median (SD) = 0.767 (1.13) vs. 1.58 (1.56), *p* = 0.048). However, in postoperative levels, no significant difference is observed between groups (median (SD) = 1.22 (2.93) vs. 1.58 (1.83), *p* = 0.315). Statistics performed by Wilcoxon rank‐sum test, comparing each high‐risk factor status group to correct. *p < 0.05. (B) Coefficients and penalty parameter illustration in the LASSO regression model. (C) Cross‐validation plot for the LASSO regression, showing the optimal value of the regularization parameter λ = 0.05376945.

Our results demonstrated a significant difference in the distribution of preoperative CD133‐positive CTC counts between the patients stratified by the presence of pathological high‐risk factors (*p* = 0.048, Wilcoxon rank‐sum test, Figure 4A). However, no significant difference in postoperative CD133‐positive CTC counts was observed between the two groups (*p* = 0.315). Therefore, preoperative CD133‐positive CTCs were chosen as a potential predictive indicator for subsequent steps in model development and analysis. Based on the preoperative CD133‐positive CTCs count, patients were subsequently classified by the existence of high‐risk factors on the basis of their preoperative CD133‐positive CTC count. Although we detected postoperative CD133‐positive CTCs, no significant changes in values or clinically relevant differences were observed at the various postoperative time points in the analysis. Therefore, the clinical value of postoperative CD133‐positive CTCs may be relatively limited.

### 3.3 Key Predictive Variables Identified Through LASSO Regression

In this study, the presence of pathological high‐risk factors is taken as the dependent variable, and each index of correlation variables is taken as the independent variable. By LASSO regression and 10‐fold cross validation, key predictive variables were selected. The optimal penalty coefficient (λ) was determined at the value that minimized the binomial deviance in the cross‐validation, ensuring the model's best predictive performance (Figure 4C). The optimum penalty coefficient λ was determined. When λ is minimum, the model exhibits the best predictive performance. Finally, six nonzero coefficients as potential predictors were screened, which were nodule characteristic, preoperative CD133‐positive CTCs, elevated serum levels of CEA, NSE, Cyfra21‐1, and SCC. The LASSO penalty coefficient path diagram is shown in Figure 4B, and the cross‐validation is shown in Figure 4C.

### 3.4 Significant Predictors Identified by Multivariate Logistic Regression Analysis

As presented in Table 2, multivariate logistic regression analysis using LASSO regression to select variables showed that nodules characteristic, elevated CEA, preoperative CD133‐positive CTCs, elevated NSE, elevated Cyfra21‐1, and elevated SCC were predictive factors for the occurrence of pathological high‐risk factors in patients. This table reports the regression coefficient (CoEF), *Z* value, *p* value, odds ratio (OR), and its 95% confidence interval (CI).

**TABLE 2:** The multivariate logistic regression analyses.

Predictor	CoEF	*Z*	*p*	OR (95% CI)
Nodules characteristic	−0.919	−2.381	0.017	0.399 (0.180–0.843)
Elevated CEA	2.180	2.081	0.037	8.850 (0.903–64.297)
Preoperative CD133‐positive CTCs	0.481	2.172	0.030	1.617 (1.025–2.494)
Elevated NSE	1.112	1.347	0.178	3.042 (0.504–14.244)
Elevated Cyfra21‐1	0.632	0.747	0.455	1.881 (0.297–8.988)
Elevated SCC	1.173	0.822	0.411	3.232 (0.110–40.03)

Abbreviations: CI, confidence interval; CoEF: coefficient; OR: odds ratio.

In summary, statistically significant factors (*p* < 0.05) filtered for the multivariate analysis included nodules characteristic (OR: 0.399, 95% CI: 0.180–0.843), elevated CEA (OR: 8.850, 95% CI: 0.903–64.297), and preoperative CD133‐positive CTCs (OR: 1.617, 95% CI: 1.025–2.494). Specifically, the presence of solid nodule features, elevated serum CEA levels, and a higher preoperative count of CD133‐positive CTCs were each significantly associated with an increased probability of harboring pathological high‐risk factors.

The model demonstrating a good model fitting, as indicated by a nonsignificant goodness‐of‐fit test yielded a *p* value of 0.8255 (Hosmer–Lemeshow test). McFadden's *R* ^2^ was 0.2091875, suggesting that approximately 20.9% of the variability was explained by the model.

### 3.5 Model Validation and Performance

**FIGURE 5:**
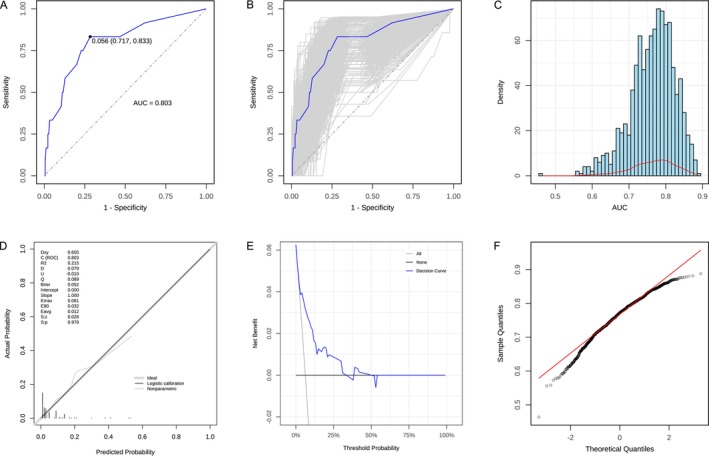
Performance evaluation of CD133‐positive CTCs in predicting pathological risk factors. (A) Receiver‐operating characteristic (ROC) curve analysis showing an area under the curve (AUC) of 0.803 for CD133 in distinguishing between low and high pathological risk factors. (B) Internal cross‐validation of the ROC curve using the bootstrap method for model validation. (C) Histogram and (F) Q‐Q plot illustrating the distribution of AUC values obtained from bootstrap resampling. (D) Calibration curve demonstrating the agreement between predicted and observed risk. (E) Decision curve analysis evaluating the net clinical benefit of the predictive model.

The model demonstrated strong predictive performance, with an area under the receiver operating characteristic curve (AUC) of 0.803 (95% CI: 0.6606–0.9445 [DeLong method]), indicating good discriminative ability (Figure 5A).

Internal validation was performed using bootstrap resampling with 1000 iterations to assess the stability and robustness of the model (Figure 5B). The normality test revealed that the AUC values followed a normal distribution (Shapiro–Wilk normality test, *p* > 0.05). In addition, the quantile–quantile plot (Figure 5F) can help depict the characteristics of the data distribution. The frequency distribution histogram (Figure 5C) shows the distribution of 1000 AUC values obtained through internal validation using the bootstrap method. The results showed that the AUC values are predominantly concentrated around 0.8, indicating the model has strong discriminant ability and stability.

Finally, a calibration curve (Figure 5D) was constructed to graphically assess the calibration of the model. Both the calibration curve and decision curve analysis (DCA) curve (Figure 5E) demonstrated that the actual results were in good agreement with the prediction probability, indicating that the model was well calibrated, and the nomogram constructed by the training set was accurate.

### 3.6 Construction of Nomogram

**FIGURE 6:**
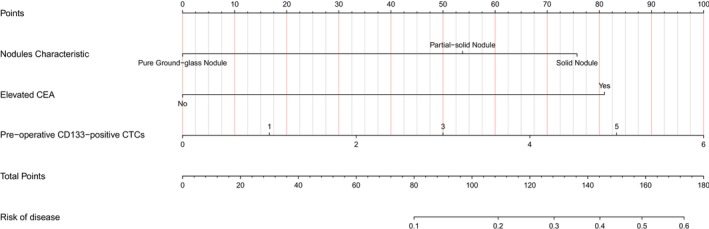
Nomogram constructed based on the independent risk factors.

A nomogram was constructed to provide a visual tool for individualized risk prediction of pathological high‐risk factors, based on the final multivariate logistic regression model (Figure 6). The results showed that the nodule characteristic (the more inclined to solid nodules), elevated CEA, and the greater number of CD133‐positive CTCs before surgery, the greater the risk of patients having pathological high‐risk factors. As illustrated, the score for each variable increases with its risk level. The total points accumulated from all variables are then mapped to a predicted probability axis, indicating the individual's risk of harboring pathological high‐risk factors. Consequently, a higher total score, derived from solid nodule features, elevated CEA, and a greater number of CD133‐positive CTCs, corresponds to a greater probability of high‐risk pathology.

### 3.7 High‐Risk Pathological Factor May Associated With PFS

**FIGURE 7:**
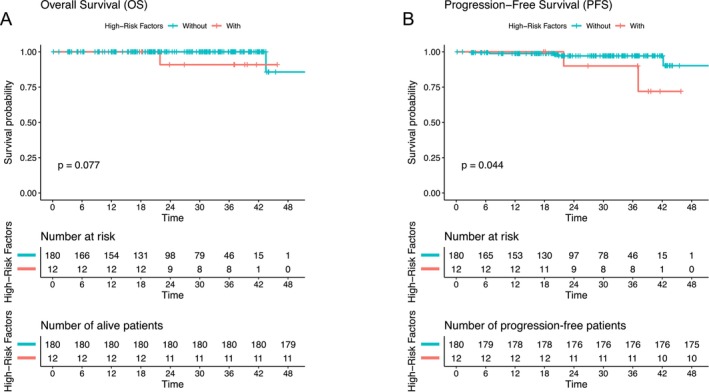
Kaplan–Meier analysis for subgroups based on the independent risk factors. This figure presents Kaplan–Meier survival curves for (A) overall survival (OS) and (B) progression‐free survival (PFS) in non‐small cell lung cancer patients, categorized based on the presence of high‐risk factors. (A) The *p*‐value from the log‐rank test is 0.077 for OS, indicating that high‐risk factors do not have a statistically significant effect on overall survival. (B) The *p*‐value from the log‐rank test is 0.044 for PFS, suggesting that high‐risk factors have a statistically significant impact on progression‐free survival.

Survival outcomes were compared between patients with and without high‐risk pathological factors. While no significant difference in OS was observed between the two groups (log‐rank test, *p* = 0.077), patients with high‐risk factors experienced a significantly worse progression‐free survival (PFS) compared to those without (log‐rank test, *p* = 0.044). Some patients in the group with high‐risk pathological factors experienced tumor recurrence, while the majority of patients in the group without high‐risk factors maintained a prolonged PFS. Kaplan–Meier plots (Figure 7) were generated to estimate and visualize the survival data in this study.

## 4 Discussion

Lung cancer is still the foremost reason for cancer‐related deaths worldwide [1]. Surgery is often an effective treatment for stage I patients of NSCLC [4]. Even so, clinical outcomes for patients can vary; postoperative recurrence and metastasis are still a risk, underscoring the significance of early and accurate diagnosis [22]. Notably, patients with stage Ib NSCLC have lower survival rates compared to those with stage Ia, likely due to the presence of pathological high‐risk factors [23, 24, 25]. Therefore, the accurate preoperative prediction of pathological high‐risk factors is crucial for developing personalized surgical plans.

For lung cancer stage I patients of NSCLC with pathological high‐risk factors, surgery alone has limited efficacy, and the variations in the extent of surgical resection may lead to varying clinical outcomes [14]. Many studies have confirmed that adjuvant chemotherapy is required to maximize survival even in stage I patients of NSCLC [26, 27]. Moreover, researchers have demonstrated that patients undergoing different resection extents experience differing survival outcomes, particularly in patients with associated pathological risk factors. It has been suggested that wedge resection may be a key factor influencing the patients' prognosis with STAS around the tumor [14]. Meanwhile, several studies have also confirmed that the presence of STAS is associated with shortened recurrence‐free survival and OS in patients of NSCLC [28, 29, 30]. Additionally, when the micro‐component exceeds 5%, the prognosis of segmental resection is significantly worse than lobectomy [31]. Therefore, noninvasive methods to predict the pathological high‐risk factors before surgery can provide an important basis for clinical decision‐making so as to optimize the surgical plan and improve the therapeutic outcomes.

This study successfully developed a preoperative noninvasive prediction model. Our results suggest that nodule characteristics, elevated CEA, and the number of CD133‐positive CTCS are potential independent predictors of pathological high‐risk factors. The model demonstrated strong discriminatory ability, as evidenced by the high AUC value (0.803) of the ROC curve. Bootstrap validation and its ROC curve further confirm that the model has stable and consistent prediction performance across different sample subsets.

The preoperative identification of pathological high‐risk factors remains clinically challenging. Nevertheless, several indicators with prognostic value have been identified in existing research. For example, the application of CT imaging technology in stage I patients of NSCLC exhibited that the overall total recurrence rate of solid nodules was significantly higher than that of patients with part‐solid nodules (17.2% vs. 3.0%, *p* < 0.001) [32]. Even in the early stage of lung cancer, the prognosis of patients with solid nodules is far worse than that of patients with subsolid nodules [33, 34]. The above findings are consistent with the results of this study; a tendency towards solid nodules is closely related to the increased risk of patients with pathological high‐risk factors.

The level of plasma CEA serves as a critical reference marker for the diagnosis, prognosis assessment, and follow‐up surveillance of lung cancer [35, 36]. Elevated CEA levels generally are associated with a higher likelihood of metastasis and recurrence in patients [37, 38, 39]. In our study, elevated CEA was also identified as an independent predictor of pathological high‐risk factors for lung cancer, further supporting its clinical relevance in risk stratification, which aligns with the results of previous research.

Furthermore, applications based on liquid biopsy techniques, such as CTC detection [40], exhibit promising prospects for noninvasive cancer monitoring. By conducting regular minimally invasive blood collections, we are able to assess the effectiveness of clinical interventions, which makes early detection of cancer or recurrence possible [41]. Notably, among patients with NSCLC, there was a high overlap (91%) between the elevated CTC detected and those with cancer metastases found on imaging 10 months later [41]. However, most current biopsy studies focus on developing prognostic models that have not provided sufficient practical guidance for surgical decision‐making or prognosis prediction, thus limiting their ability to improve clinical outcomes.

CSCs are pivotal in driving tumor proliferation, metastasis, and recurrence due to their strong ability for self‐renewal and differentiation [42]. Among them, CD133 serves as a key marker for various cancer CSCs, with its expression level negatively correlated with the degree of cell differentiation, which is crucial for maintaining stem cell properties and driving tumor progression [43, 44]. Among all the blood samples in this study, only a small number of samples could not undergo final analysis due to technical issues during the collection or detection process. The overall failure rate of detection was low. According to our experience and this cohort, the positive rate of CTC in patients with malignant pulmonary nodules is about 76.3%. As a diagnostic marker, CD133 is widely used for stratifying and assessing cancer patients, particularly in brain, colon, and prostate cancers, where it has demonstrated good prognostic value [45, 46, 47, 48, 49]. In the detection methods of CSCs, CTC analysis is dominant [50]. Our previous studies demonstrated that TBCD technology has excellent performance in the diagnosis of pulmonary nodules [21], but it is still insufficient in the prediction of pathological high‐risk factors. Therefore, we used TBCD technology to assess stem cell markers in preoperative CTCs, aiming to establish a diagnostic model to aid in precise surgical decision‐making and optimize patient prognosis. Our results indicate that TBCD combined with anti‐CD133 antibody technology enables specific identification of CD133+ CTCs, potentially further enhancing patient treatment outcomes and survival quality. During the CTC detection process, we ensured the accuracy and reliability of the results by using a standardized sample processing protocol, for example, setting exclusion criteria (such as filtering low‐quality samples). For suspected low‐quality or abnormal samples, we performed retesting or recollected blood samples. Additionally, white blood cell interference was one of the challenges we encountered in some cases, particularly during the acute infection phase of the patient, where the high number of white blood cells in the blood could affect the fluorescence labeling recognition of tumor cells. To minimize this interference, we would recollect blood samples from the patients to ensure the accuracy of the detection results.

The results of survival analysis in this study indicate that in stage I NSCLC patients, no significant difference was observed in OS between the groups. This may be attributed to the relatively high survival rate of stage I NSCLC patients and the potential positive impact of surgical intervention. However, a significant difference in PFS was observed between the two groups, with some patients in the high‐risk pathological factor group experiencing recurrence. In conjunction with other studies, these findings suggest that pathological high‐risk factors may be associated with an increased risk of tumor recurrence, warranting further attention. Therefore, more personalized treatment and monitoring strategies should be developed for patients with pathological high‐risk factors, not only during the surgical planning phase but also for postoperative monitoring, to optimize their prognosis. It should be noted that the follow‐up period was relatively short, and therefore the clinical implications may be limited.

It is noteworthy that the present study still harbors several limitations. Firstly, the underlying mechanisms of lung cancer metastasis progression remain unexplored. Secondly, the model, based on data from a specific patient cohort at a single medical center, may lack universal applicability across diverse populations. Given the limited study timeframe, clinical data may be biased, affecting model generalizability. Crucially, despite the promising predictive performance demonstrated by CD133‐positive CTCs and other selected indicators, the current model may not have fully incorporated other potential biomarkers or clinical variables, indicating room for improvement. Therefore, external validation in different patient cohorts is crucial to verify the model's effectiveness and reliability in various scenarios. In our analysis of PFS differences, we did not account for other potential confounding factors, which may affect the accuracy and interpretation of the results. Subsequent studies can further focus on the changes in CTCs at different time points during the perioperative period, or combine other molecular markers to improve the accuracy of prognostic assessment. By incorporating more follow‐up and survival information in larger sample sizes and multicenter studies, the potential role of CD133‐positive CTCs in predicting the risk of postoperative recurrence may be better elucidated.

## 5 Conclusion

In conclusion, this study successfully established a preoperative, noninvasive predictive model that integrates CD133‐positive CTCs with key clinical characteristics to assess the pathological high‐risk factors in patients with NSCLC. Statistically validated, it shows robust accuracy and utility, aiding clinical decisions. Future research should broaden its applicability and validate across diverse populations.

## Author Contributions

**Huandong Huo:** conceptualization, formal analysis, writing – original draft. **Xiaoli Zhang:** conceptualization, formal analysis, investigation, validation, writing – review and editing. **Qi Zhang:** investigation. **Zhuoheng Lv:** investigation. **Peipei Xie:** investigation. **Kaitai Zhang:** supervision, funding acquisition. **Wen Zhang:** writing – review and editing. **Yousheng Mao:** conceptualization, supervision, funding acquisition.

## Ethics Statement

The research protocol and consent form were reviewed and approved by the Medical Ethics Committees of National Cancer Center/National Clinical Research Center for Cancer/Cancer Hospital, Chinese Academy of Medical Sciences and Peking Union Medical College.

## Conflicts of Interest

The authors declare no conflicts of interest.

## Data Availability

The data that support the findings of this study are available on request from the corresponding author. The data are not publicly available due to privacy or ethical restrictions.
